# Sources of Uncertainty in a DVM-Based Measurement System for a Quantized Hall Resistance Standard

**DOI:** 10.6028/jres.099.018

**Published:** 1994

**Authors:** Kevin C. Lee, Marvin E. Cage, Patrick S. Rowe

**Affiliations:** National Institute of Standards and Technology, Gaithersburg, MD 20899-0001

**Keywords:** digital voltmeter, DVM method, electrical metrology, electrical reference standards, quantized Hall resistance, quantum Hall effect, resistance calibration

## Abstract

Transportable 10 kΩ standard resistors have become fairly widespread in industrial, university, and government standards laboratories because of their low temperature coefficient of resistance, case of transportation, and convenient value. The values of these resistors, however, tend to drift with time, requiring periodic recalibration against an invariant standard such as the quantized Hall resistance. The availability of a simple, inexpensive measurement system for calibrating 10 kΩ resistors against such an invariant standard would be of great benefit to primary standards laboratories. This paper describes a simple automated measurement system that uses a single, high accuracy, commercially available digital voltmeter (DVM) to compare the voltages developed across a 10 kΩ standard resistor and a quantized Hall resistor when the same current is passed through the two devices. From these measurements, the value of the 10 kΩ standard resistor is determined. The sources of uncertainty in this system are analyzed in detail and it is shown that it is possible to perform calibrations with relative combined standard uncertainties less than 1×10^−7^ (0.1 ppm).

## 1. Introduction

Resistors composed of coils of wire wound around suitable forms have been used as standards of resistance for many years [1]. Such devices arc even today widely used as working standards of resistance by primary and secondary standards laboratories in industry, university, and government. Due to aging of the wire and other effects, however, the values of these resistors tend to drift with time, requiring periodic recalibration against a known standard. Because wire-wound resistors drift with time, many national standards laboratories have adopted a standard of resistance based on the quantum Hall effect [2]. When a sample containing a thin, two dimensional conducting layer known as a two-dimensional electron gas (2 DEG) is cooled to liquid helium temperatures in the presence of a very strong magnetic field, the resistance of the device becomes quantized, assuming well defined values given by

$$R_{H}(i)=\frac{V_{H}(i)}{I}=\frac{h}{ie^{2}}\equiv \frac{R_{K}}{i}$$ (1)

where *V*_H_ is the voltage across the Hall device, *I* is the current through the device, *h* is the Planck constant, *e* is the elementary charge, *i* is an integer, and *R*_K_, the von Klitzing constant, has been defined by international agreement to be exactly 25 812.807 Ω for the purposes of practical electrical metrology [3]. The resistance is time invariant, and is, under appropriate conditions of measurement, independent of the measurement conditions, such as current, temperature, and magnetic field. Because of these properties, the quantized Hall resistance, by international agreement, has been used since January 1, 1990 as a practical representation of the ohm.

The measurement systems in use at national standards laboratories are quite complex and are capable of achieving relative combined standard uncertainties [4] of 1 × 10^8^ (or 0.01 ppm where 1 ppm = 1×10^6^) or less [5]. Many government and industrial standards laboratories do not need such small uncertainties in their work. Indeed, the uncertainty required by many laboratories is sufficiently large that the drifts in the values of wire-wound artifact standards are less than their measurement resolution and are therefore ignored. Such laboratories are well served by wire-wound artifacts which can be sent periodically to national standards laboratories to be calibrated or to participants in NIST’s Measurement Assurance Program, or MAP. Generally, the relative combined uncertainty achieved in the MAP is about 0.1 ppm to 0.2 ppm.

Some laboratories, however, require smaller uncertainties. At such levels, drifts of the values of wire-wound artifacts require that they be frequently recalibrated. For these laboratories, the availability of a simple and inexpensive invariant standard of resistance would be of great benefit. The fairly recent introduction of high accuracy commercial digital voltmeters (DVMs) with 8 1/2 digit resolution has made it possible to conceive of a simple and fairly inexpensive measurement system [6] that would enable government and industrial standards laboratories to perform calibrations of their wire-wound resistors directly against a quantized Hall resistor with uncertainties of 0.1 ppm or less.

Such a calibration system has three distinct parts to it: a quantized Hall resistance device, a cryogenic system in which a superconducting solenoid and the Hall device are cooled to liquid helium temperatures, and a measurement system for comparing the standard resistor to this quantized Hall resistor. While the selection of the sample and cryogenic system are beyond the scope of this paper, a few words must be said about them, for they affect the design of the measurement system [7]. The sample and the cryogenic system must be such that the conditions for accurate measurement of the Hall resistance are met [8]. The conditions pertinent to this discussion are that the plateaus in the Hall voltage extend over as broad a range of magnetic field as possible, and that the voltage drop along the sample in the direction of the current flow, *V_x_*, be as small as possible under the conditions of measurement.

While in theory any Hall plateau [any value of *i* in Eq. (1)] can be used for resistance calibration, in practice, the plateaus corresponding to higher values of *i* (i > 4) tend, except in unusual cases, to have values of *V_x_* which are unacccptably large for precision resistance calibrations [9]. In general, the plateaus corresponding to lower values of *i* (*i*=4,2,1) occur at higher magnetic fields, are broader, and have values of *V_x_* that are small enough to permit accurate resistance calibrations. The value of magnetic field at which any given plateau occurs is a function of the electron concentration in the 2 DEG, which in turn is a function of the design of the sample: samples can be designed to exhibit the *i*=4 plateau, for example, at very low fields of only a few tesla, or very high fields of 10 T or more. In choosing a sample design, one must balance several factors: if the plateau occurs at lower field, it will be more accessible with an inexpensive magnet and cryogenic system, but it will be narrower, and possibly *V_x_* may be too large; on the other hand, if the plateau occurs at a high magnetic field, the plateau will be broader, *V_x_* will be smaller, but the cryogenic system and magnet will have to be much larger, and consequently more expensive. It is the opinion of the authors that the optimum sample exhibits the *i*=4 plateau [*R*_H_(4)=6 453.201 75 Ω] in a magnetic field range of 4.5 T to 6.5 T, and the *i*=2 plateau between 9.0 T and 13.0 T. These fields are easily attainable with commercially available superconducting magnets. The discussion of the measurement system in this paper therefore assumes that the resistance of the Hall device, *R*_H_(*i*), will be 6 453.20175 Ω, I2 906.403 5 Ω, or 25 812.807 Ω, corresponding to the *i*=4, 2, or 1 plateau.

## 2. Description of Measurement System

**Fig. 1:**
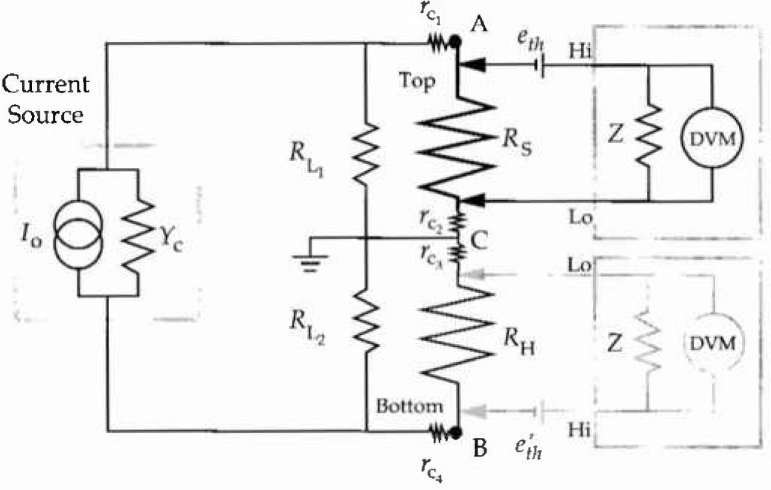
DVM-based measurement system for comparing wire-wound standard resistors (*R*_S_) with a quantized Hall resistor (*R*_H_). $R_{L_{1}}$, and $R_{L_{2}}$ are the net leakage resistances to ground of the cables, current source, resistors, and the rest of the measurement system. The contact resistances are denoted by $r_{c_{1}},\ldots ,r_{c_{4}}$. The voltage across each resistor is measured with the digital voltmeter (DVM). The thermal voltages generated at the connections between the DVM and the resistors are denoted by *e*_th_ and *e*′_th_. Z is the internal impedance of the DVM and *Y_c_* is the Norton equivalent admittance of the current source.

This measurement system is shown schematically in Fig. 1. The standard resistor to be calibrated (*R*_S_) is placed in series with the Hall device (*R*_H_). A constant current is passed through both resistors, and the potential difference across each resistor is measured with the DVM. All measurements of resistance are four-terminal measurements. The current source and DVM are connected to the resistors using switches, so that the direction of current flow can be easily reversed, and the meter can be connected to either of the resistors. The measurements are performed in the order described by Marullo-Reedtz and Cage [5]. The potential drop *V*_R_ across the standard resistor *R*_S_ is first measured with the current flowing in the “positive” direction. The current direction is then reversed, and the potential drop across the standard resistor is measured again. These two measurements are repeated in the reverse order, to give a set of four values:

$$V_{R}(+I),\;V_{R}(-I),\;V_{R}(-I),\;V_{R}(+I).$$ (2)

The DVM is then connected to the Hall device, and this same sequence of four measurements is made on it twice. Finally, the standard resistor is measured again. The entire sequence of measurements is:

$$(1)\;V_{R}(+I),\;V_{R}(-I),\;V_{R}(-I),\;V_{R}(+I)\Rightarrow \langle V_{R1}\rangle$$ (3a)

$$(2)\;V_{H}(+I),\;V_{H}(-I),\;V_{H}(-I),\;V_{H}(+I)\Rightarrow \langle V_{H2}\rangle$$ (3b)

$$(3)\;V_{H}(+I),\;V_{H}(-I),\;V_{H}(-I),\;V_{H}(+I)\Rightarrow \langle V_{H3}\rangle$$ (3c)

$$(4)\;V_{R}(+I),\;V_{R}(-I),\;V_{R}(-I),\;V_{R}(+I)\Rightarrow \langle V_{R4}\rangle$$ (3d)

In all, a group of sixteen measurements is taken. The four individual values of each of these sets is averaged to yield a group of four values shown at the right. It will be shown later that this eliminates the effects of thermal voltages that are either constant or vary linearly with time. Finally, the two voltage drops across the standard resistor, 〈*V*_R1_〉 and 〈*V*_R4_〉 are averaged, as are the two measurements of the voltage drop across the Hall resistor, 〈*V*_H2_〉 and 〈*V*_H3_〉:

$$\langle V_{R}^{\mathrm{TOP}}\rangle =\frac{\langle V_{R1}\rangle +\langle V_{R4}\rangle }{2},$$ (4a)

$$\langle V_{H}^{\mathrm{BOT}}\rangle =\frac{\langle V_{H2}\rangle +\langle V_{H3}\rangle }{2}.$$ (4b)

The above measurement sequence is then repeated with the positions of the Hall resistor and the standard resistor interchanged: i.e., the Hall resistor is connected in the top position of the circuit in Fig. 1, and the standard resistor is placed in the bottom position. The average voltages in Eqs. (4a) and (4b) are then computed for this interchanged position, yielding $\langle V_{R}^{\mathrm{BOT}}\rangle$ and $\langle V_{H}^{\mathrm{TOP}}\rangle$.

In theory, if the current through the two resistors is constant,

$$\frac{\langle V_{R}^{\mathrm{TOP}}\rangle }{\langle V_{H}^{\mathrm{BOT}}\rangle }=\frac{\langle V_{R}^{\mathrm{BOT}}\rangle }{\langle V_{H}^{\mathrm{TOP}}\rangle }=\frac{R_{S}}{R_{H}}.$$ (5)

In practice, the situation is complicated greatly by a large number of factors, including thermally generated voltages, leakage resistances, contact resistances, instabilities in the current source, noise in the voltmeter, etc. It is shown in Sec. 3 that by application of the measurement procedure outlined above, the effects of several of these factors can be eliminated or minimized. Other factors cannot be eliminated by design of the measurement system, and their effects must be independently minimized. Some of these factors contribute to the error of the determination of the value of the standard resistor. At this point it should be noted that the value of the standard resistor *R* is usually expressed as a deviation from its nominal value, denoted $R_{S}^{\mathrm{nom}}$:

$$\left(\frac{R_{S}-R_{S}^{\mathrm{nom}}}{R_{S}^{\mathrm{nom}}}\right)=\frac{R_{S}}{R_{S}^{\mathrm{nom}}}-1.$$

This ratio can be expressed in terms of the voltage ratios in Eq. (5) as:

$$\frac{R_{S}}{R_{S}^{\mathrm{nom}}}-1=\frac{\langle V_{R}\rangle }{\langle V_{H}\rangle }\frac{R_{H}}{R_{S}^{\mathrm{nom}}}-1.$$

As a result, the correction factors and uncertainties in the ratio of the voltages in Eq. (5) must be divided by the ratio $\frac{R_{S}^{\mathrm{nom}}}{R_{H}}$ in order to obtain the correction factors and uncertainties in the deviation of the resistor from its nominal value.

In this paper, relationships between the magnitudes of the various effects and the resulting error in the value of the standard resistor are determined, it is shown in Sec. 3 that if certain limits are placed on the magnitudes of the various systematic effects, the magnitudes of the factors required to correct for their effect on the value of the standard resistor are significantly less than the uncertainty in the value of the standard resistor due to random effects, and the corrections can be neglected while still maintaining a relative combined standard uncertainty of 0.1 ppm. In Sec. 4, the uncertainties due to random effects, such as nonlinear drifts in the output of the current source and in the gain and offset of the DVM, etc., arc estimated. Limits are derived for the maximum values of these factors to ensure that the relative combined standard uncertainty in the value of the standard resistor is of the order of 0.1 ppm. It is shown that an uncertainty of 0.1 ppm or less can be obtained when calibrating resistors with *R_S_* as much as a factor of 4 different from the quantized Hall resistance, *R*_H_(*i*) [this holds true whether the *i* = 1, 2, or 4 plateau is used for *R*_H_(*i*) i.e., whether the value of *R*_H_(*i*) is 25 812.807 Ω, 12 406.403 5 Ω, or 6 453.201 75 Ω].

## 3. Corrections Arising From Systematic Effects

Systematic effects that can contribute significantly to the error of the determination of the value of the standard resistor arc associated with four main parts of the measurement system. These are:

- the wires, cables, and switches used to connect the Hall resistor and the standard resistor;
- the Hall device and standard resistor;
- the current source used to supply the current through the resistors; and
- the DVM used to measure the voltages across the two resistors.

The magnitudes of the errors arising from each of these parts of the measurement system are estimated in this section.

### 3.1. Measurement System

In the measurement system shown in Fig. 1, the Hall resistor and the standard resistor to be calibrated are connected in series, and are connected, by means of cables and switches, to a current source. In principle, the same current flows through each resistor, so the ratio of the voltage measured by the DVM across the standard resistor and the Hall resistor should equal the ratio of the values of the resistors. In practice, however, there are thermally generated voltages in the wires, switch contacts, and various connections in the circuit which cause the measured voltages to differ from the actual voltage drops across the resistors. It is shown in Sec. 3.1.1 that the averaging technique described in Eq. (3) above eliminates the effect of thermal voltages that are either constant or vary linearly with time. Variable contact resistances in the switches connecting the resistors to the current source can cause variations in the current supplied by the current source. In Sec. 3.1.2, an upper limit is derived for the magnitude of the permissible variations in the contact resistances. Leakage currents in the cables, switches, and other components of the system can also result in errors in the measurements of the voltages. The errors due to the leakage resistances can be corrected for, as described in Sec. 3.1.3.

#### 3.1.1 Thermal Electromotive Forces

Thermally generated voltages in the switch contacts, the wires, and the connections to the DVM result in erroneous determinations of the voltage drops across the resistors in the circuit. These thermal voltages are denoted by *e*_th_ and *e*′_th_ in Fig. 1, and arise when there are temperature variations between various parts of the circuit and when there are junctions between dissimilar materials, such as in switches or at solder connections. Thermal voltages are therefore generated at the connections between the current source and each resistor, and at the connections between the DVM and each resistor. The thermal voltages generated in the contacts to the current source add to the current produced by the current source. As is shown in the next section, if the equivalent admittance of the current source (*Y_c_* in Fig. 1) is low enough, the current source will adjust its output to maintain a constant current through the circuit, and these thermal voltages have no effect on the measurement. Thermally generated voltages in the contacts to the DVM, on the other hand, are significant, and must be corrected for. Since the thermal voltages are a function of the temperature differences in the circuit, they do not change sign when the current is reversed. Thus, if one averages the voltage across one of the resistors measured with the current in one direction and the voltage across the same resistor obtained with the current in the opposite direction, the thermal voltage docs not contribute to the average. Specifically, if the Hall resistor is in the BOTTOM position of the circuit of Fig. 1, the voltage drop across it with the current flowing in one direction (which will be denoted as the “positive” direction) is

$$V_{H}^{\mathrm{BOT}}(+I)=\frac{+I}{G_{L_{2}}+G_{H}}+e_{\mathrm{th}}^{\prime },$$ (6)

where $G_{L_{2}}=1/R_{L_{2}}$ is the leakage conductance in parallel with the Hall resistor (described in more detail in Sec. 3.1.3) and *G*_H_ = 1/*R*_H_ is the Hall conductance. With the current flowing in the opposite direction (denoted as the “negative” direction) one obtains

$$V_{H}^{\mathrm{BOT}}(-I)=\frac{-I}{G_{L_{2}}+G_{H}}+{e^{\prime }}_{\mathrm{th}}.$$ (7)

The average value of the voltage across the Hall resistor is then independent of the thermal voltage:

$$\langle V_{H}^{\mathrm{BOT}}\rangle =\frac{1}{2}\;\left[V_{H}^{\mathrm{BOT}}(+I)-V_{H}^{\mathrm{BOT}}(-I)\right]=\frac{I}{G_{L_{2}}+G_{H}}.$$ (8)

It has been assumed that the magnitudes of the thermal voltages are the same and independent of the current direction. Practically, the current is reversed using switches, but the connections between the resistor and the DVM are not broken when the direction of the current is changed. Thus, the thermal voltages in the DVM contacts should not change when the current is reversed, and the assumption that the thermal voltages remain constant or vary linearly with time during the measurements should be a good one. It is also important to note that it was assumed that the current does not drift with time. This is discussed in more detail in Sec. 3.2.

#### 3.1.2 Contact Resistances

Contact resistances occur at all of the junctions in the system, including solder connections, switches, and other connectors in the circuit. In addition, the ohmic contacts to the 2 DEG in the quantized Hall device also exhibit contact resistance. Voltages develop across only those contacts that have current flowing through them. The effects of the resistances of the contacts between the resistors and the DVM arc therefore minimized by making 4-terminal measurements of the voltages across each resistor, i.e., the voltage across a resistor is measured between two terminals that are separate from the terminals that carry the current.

The contact resistances $r_{c_{1}},\ldots ,r_{c_{4}}$ between the resistors and the current source shown in Fig. 1 change the total resistance of the circuit, but if they are constant and reproducible, as would be the resistances at solder connections, then it is apparent that they have no effect on the measurement. The resistances of the contacts in the switches connecting the resistors to the current source may change, however, for the switch contacts are constantly being opened and closed. If these contact resistances are *reproducible*, then they also have no effect on the total measurement. To see this, consider the case where the total contact resistance for one current direction always differs by an amount δ*Z* from that for the opposite current direction. If the equivalent admittance of the current source is zero, then obviously this has no effect. In practice, however, the admittance of the current source is nonzero, and this difference in contact resistance changes the total load impedance, and slightly affects the current through the resistors by an amount δ*I*_L_. In this case, the average in Eq. (8) is multiplied by the factor

$$\left(1+\frac{\delta I_{L}}{2I_{L}}\right),$$ (9)

where *I*_L_ is the current through the resistors. If the difference δ*Z* in the contact resistances is reproducible, then the averages of both the voltages across the Hall resistor and the standard resistor are multiplied by this same factor, and it cancels when ratios of the voltages are taken (as described in Sec. 2, and in more detail in Sec. 3.1.3).

If the contact resistances of the switches vary in a random, uncorrelated, and irreproducible manner every time they are opened and closed, each voltage has a different correction factor of the form given by Eq. (9). These correction factors do not cancel when voltage ratios are taken. As a result, it is necessary to keep δ*I*_1_/*I*_L_ of the order of 10^−8^ or less to assure uncertainties of 1 part in 10^7^ in the determination of the value of the standard resistor. This requirement places a limit on the variation in contact resistance δ*Z* which depends on the admittance of the current source: the smaller the admittance, the larger the permissible variations in δ*Z.* Specifically, if the admittance of the current source is *Y_c_*, it can be shown that [10]^1^

$$\frac{\delta I_{L}}{I_{L}}=\frac{-Y_{c}\delta Z}{\left[1+(R_{H}+R_{S})Y_{c}\right]}.$$ (10)

If the admittance of the current source is about 50 μS, *R*_H_(*i*−4) = 6453.201 75 Ω, and *R*_S_ = 10 kΩ,

$$\frac{\delta I_{L}}{I_{L}}=2.7\times 10^{-5}\delta Z,$$

and δ*Z* must be less than 0.36 mΩ for δ*I*_L_/*I*_L_ to be less than 10^−8^. If a low admittance current source is used, δ*Z* can be larger, but in general, the contact resistances in the switches and contacts of the circuit should be kept very small, and should be reproducible to within a few tenths of a milliohm, in order for them not to affect the accuracy of the measurement. This should not be difficult if care is taken to ensure that all of the contact surfaces are very clean and not covered with a thin oxide or a layer of organic contaminant.

The problem posed by the resistances of the contacts to the quantized Hall device is a vastly more subtle one. As described above, the *difference* between the resistances of the current carrying contacts to the 2 DEG with forward and reverse directions of current must be less than a few tenths of a milliohm. It is very difficult to produce contacts to the 2 DEG with such small contact resistances [11], but fortunately, the actual contact resistances need not be this low: the important point is that the contact resistances must be independent of the direction (and magnitude) of the current. Nevertheless, it is necessary that the current carrying contacts have contact resistances of less than 10 mΩ, or they generate substantial amounts of noise which prevents accurate resistance comparisons. One would think that the resistance of the contacts used to measure the Hall potential are unimportant, for no current flows through them. These contact resistances, however, must also be in the range of a few milliohms, for reasons which arc beyond the scope of this paper [7, 12].

#### 3.1.3 Leakage Resistances

Leakage resistances arise from the noninfinite resistance of the electrical insulations used in constructing the system. As a result, the leakage resistance is distributed throughout the system: there are contributions from the current source, the cables, the DVM, and even the standard resistor and the wires leading to the quantized Hall resistor. The leakage resistances from the high terminal of the current source, the current reversal switch, the cables, the standard resistor, and the DVM have been combined in the idealized “leakage resistance” $R_{L_{1}}$, shown in Fig. 1. The leakage resistances from the tow terminal of the current source, cables, etc., and the quantum Hall resistor have been combined in the idealized “leakage resistance” $R_{L_{2}}$. The circuit is grounded between the Hall resistor and the standard resistor to minimize the effects of the leakage resistance between the low terminal of the DVM and ground: the low terminal of the DVM is always connected to this point throughout the entire measurement sequence. The high terminal is alternately connected to point A in Fig. 1 to measure the standard resistor, or point B to measure the Hall resistor. If it can be assumed that the leakage resistances $R_{L_{1}}$, and $R_{L_{2}}$ remain constant throughout the measurement cycle, and that the Hall resistor and the standard resistor have nominally the same values, it can be shown that the error due to leakage resistance is eliminated by averaging the ratio of the voltage across the standard resistor to the voltage across the Hall resistor with the resistors in the standard configuration shown in Fig. 1 and the same ratio obtained when the positions of the resistors in the measurement circuit are interchanged. With the resistors connected as shown in Fig. 1 (the standard position), the average voltages across the resistors [as determined from Eq. (8)] are:

$$\langle V_{R}^{\mathrm{TOP}}\rangle =\frac{I}{G_{L_{1}}+G_{S}}$$ (11a)

$$\langle V_{H}^{\mathrm{BOT}}\rangle =\frac{I}{G_{L_{2}}+G_{H}},$$ (11b)

where *G* = 1/*R* for each of the resistors in Fig. 1. The ratio of these two voltages is then

$$\begin{array}{c} \frac{\langle V_{R}^{\mathrm{TOP}}\rangle }{\langle V_{H}^{\mathrm{BOT}}\rangle }=\frac{G_{L_{2}}+G_{H}}{G_{L_{1}}+G_{S}}\\ =\frac{G_{H}}{G_{S}}\frac{1+\frac{G_{L_{2}}}{G_{H}}}{1+\frac{G_{L_{1}}}{G_{S}}}=\frac{G_{H}}{G_{S}}\left(1+\frac{G_{L_{2}}}{G_{H}}-\frac{G_{L_{1}}}{G_{S}}\right). \end{array}$$ (12)

If it is assumed that the leakage resistances are independent of the positions of the resistors, the positions of the Hall resistor and the standard resistor can be usefully interchanged. In this case, the Hall resistor is in parallel with $R_{L_{1}}$ and the standard resistor is in parallel with $R_{L_{2}}$. The ratio of Eq. (12) is then

$$\frac{\langle V_{R}^{\mathrm{BOT}}\rangle }{\langle V_{H}^{\mathrm{TOP}}\rangle }=\frac{G_{L_{1}}+G_{H}}{G_{L_{2}}+G_{S}}\approx \frac{G_{H}}{G_{S}}\left(1+\frac{G_{L_{1}}}{G_{H}}-\frac{G_{L_{2}}}{G_{S}}\right).$$ (13)

Averaging Eqs (12) and (13) yields:

$$\begin{array}{c} \frac{1}{2}\left(\frac{\langle V_{R}^{\mathrm{TOP}}\rangle }{\langle V_{H}^{\mathrm{BOT}}\rangle }+\frac{\langle V_{R}^{\mathrm{BOT}}\rangle }{\langle V_{H}^{\mathrm{TOP}}\rangle }\right)\\ =\frac{G_{H}}{G_{S}}\left(1+\frac{G_{L_{1}}+G_{L_{2}}}{2G_{H}}-\frac{G_{L_{1}}+G_{L_{2}}}{2G_{S}}\right). \end{array}$$ (14)

If the standard resistor has exactly the same value as the Hall resistor, then the last two terms on the right side of Eq. (14) cancel, and the leakage resistances have no effect. If the resistors are not equal in value, then there is a small correction factor. If the leakage resistances change when the current is reversed or the resistors arc interchanged, then the above analysis does not hold. Even in this case, however, the error in the determination of the value of the standard resistor caused by the leakage resistances will be of me order of me ratio of the leakage conductance $G_{L}=G_{L_{-1}}+G_{L_{-2}}$ to the conductance of the larger of the two resistances *R*_H_ or *R*_S_. Typically, cables insulated with PTFE Teflon, and carefully constructed current sources will have leakage resistances greater man about 10^12^ Ω (*G*_L_ < 10^−12^ S). If *R*_S_ = 10 kΩ. and *R*_H_ = 6 453.201 75 Ω or 12 906.403 5 Ω, the correction to the value of me standard resistor will be of the order of 0.003 ppm, which is more than an order of magnitude less man the uncertainty due to random effects, and can therefore be neglected. For resistance ratios of 4 or more, as would result from the comparison of a 6 453.201 75 Ω and a 25 812.807 Ω resistor, however, the correction to the value of the standard resistor can be as large as 0.02 ppm, which is comparable to the uncertainty due to random effects and cannot be neglected. In this case, a correction factor can be estimated by measuring the leakage resistance between the point C in Fig. 1 and earth which will be approximalely equal to $1/(G_{L_{1}}+G_{L_{2}})$ (in Fig. 1 point C is connected to earth, but for this leakage resistance measurement this connection must be removed).

The uncertainty associated with the assessment of this leakage resistance, however, will be quite large, possibly as large as the correction factor itself, so one can treat this correction factor as a component in the assessment of the combined uncertainty. If lower uncertainties are required, however, it will be necessary to increase the leakage resistance of the system another order of magnitude, something which is quite difficult to do.

### 3.2. Current Source

Several factors determine the optimum current for diese measurements. The higher the current, the larger the voltages across me resistors, and me smaller the averaging tíme required to obtain voltage measurements with a given experimental standard deviation of the mean. Too large a current, on the other hand, can cause self-heating of me standard resistor, which will change its value and, more importantly, can cause breakdown of the dissipationless current flow in the Hall device, rendering it unusable for resistance calibrations [13]. Typically, currents between 10 μA and 50 μA satisfy diese conditions. It should be noted that when performing calibrations of 10 kΩ resistors against a quantized Hall resistor, it is the maximum current that the quantized Hall device can sustain without breaking down that limits the maximum current at which measurements can be performed. Generally, this critical current is far below the current at which even a typical 10 kΩ resistor would start to show self-healing effects.

It should be recognized, however, mat a reduction in averaging time due to a higher current is only realized if the primary factor limiting the accuracy of the voltage measurement is the signal-to-noise ratio and *not* the resolution of the meter. If the primary limiting factor is the resolution of the meter, then the benefits of increasing the current are somewhat limited. For example, if the resolution of the meter is 10 nV on the 100 mV range, then one benefits from choosing a current that produces a voltage near the top of this range, as in this case the voltage measurement has the minimum relative uncertainty. Increasing the current so that the voltage is so large that the meter must use the next range, e.g., the 1 V range, however, may not result in any benefits if the resolution of the 1 V range is 100 nV. In this case, the relative uncertainty of the voltage measurement will be the same or worse than at the lower current. In practice, some benefit may be obtained by using the 1 V range in this example, for the internal DVM noise is often less on the higher voltage ranges than on the 100 mV range, so lower uncertainties in the measurement of the voltage can be achieved with shorter measurement times, even though the resolution of the meter Is poorer on the 1 V range.

The method for determining the ratio of the value of the standard resistor to the Hall resistance described in Sec. 2 assumes that the current through the resistors is constant during the time that the voltage measurements are made. Any variations in the current, such as drifts or noise, will appear as drifts or noise in the voltages across the resistors and will affect the accuracy of the calibration of the standard resistor. In order to obtain a relative combined standard uncertainty in the calibration of 10^−7^, it is necessary to keep the noise in the current source and nonlinear deviations in the current below 0.01 ppm; for currents between 10 μA and 50 μA, this dictates that the current variations be less than 0.1 pA to 0.5 pA. Such low noise levels are rather difficult to achieve with active current sources containing operational amplifiers, transistors, or other solid state components which usually have noise levels of the order of a few ppm, and are therefore generally unacceptable for use with this method. Current sources made using mercury batteries and current-limiting wire-wound resistors are capable of meeting these stringent requirements, even though the output of these current sources tends to decrease with time in a predictable, linear manner.

Fortunately, such stable linear drifts in current do not affect the measurements if the measurement sequence described in Sec. 2 is used. If the current decreases at a constant rate *B*, and the current at the beginning of the first measurement is *I*, then the current at a time *t* after the first measurement was begun is

I(t)=I−Bt. (15)

If each individual voltage measurement described in Sec. 2 takes a time Δ*t*, then the current at the start of each voltage measurement in the group is given by me expression in column 2 of Table 1. As described above in Sec. 2, the first four voltage measurements (of the standard resistor with positive and negative current) are averaged to eliminate thermal voltages, yielding the value shown in the third column of Table 1. The average voltages across the standard resistor are then averaged, as are the average voltages across the Hall device, as described in Sec. 2, Eq. (4a), to give

$$\langle V_{R}^{\mathrm{TOP}}\rangle =\frac{\langle V_{R1}\rangle +\langle V_{R4}\rangle }{2}\approx R_{S}\left[I_{0}-\left(\frac{30}{4}B\;\Delta t\right)\right].$$ (16a)

$$\langle V_{R}^{\mathrm{BOT}}\rangle =\frac{\langle V_{H2}\rangle +\langle V_{H3}\rangle }{2}\approx R_{H}\left[I_{0}-\left(\frac{30}{4}B\;\Delta t\right)\right].$$ (16b)

The effective current is the same in both of these equations, and is eliminated when the ratio of *V*_H_ to *V*_R_ is taken. Note that the effective current in both of these equations is the current at the exact mid-point of the measurement cycle.

### 3.3 Digital Voltmeter (DVM)

The quality of the digital voltmeter used to measure the voltages across me resistors is the ultimate factor limiting the accuracy of this technique. In order to obtain resistance calibrations with relative combined standard uncertainties of 10^−7^, the DVM must be capable of measuring voltages with uncertainties about a factor of 5–10 less than this. If a 20 μA current is used, the voltages across the resistors will be of the order of 200 mV, and the DVM must be capable of resolving voltages of the order of 0.2×10^−7^ V, or 20 nV [14]. Commercial DVMs are now available from several manufacturers that have such a high resolution. In addition to the high resolution, however, the DVM must have very high accuracy, a high degree of linearity, a high input impedance, high stability, and very low noise. Commercial 8 1/2 digit multimeters from several manufacturers are on the market that meet these specifications. In this section, various systematic effects associated with the DVM that contribute to the total uncertainty arc analyzed. In Sec. 3.3.1, a correction term accounting for offsets and nonlinearities in the response of the DVM to applied voltages is derived. In Sec. 3.3.2, the effect of the small current source between the input terminals of the DVM is considered. The effect of noninfinite input impedance is considered in Sec. 3.3.3.

While this measurement system is quite similar to potentiometric measurement systems achieving smaller ultimate uncertainties [5], the accuracy, range, resolution, and linearity requirements on the DVM used with this system are greater. In the Potentiometrie measurement systems, a potentiometer is used to cancel the Hall voltage, so the detector is only used to measure very small differences between the Hall voltage and the voltage drop across the standard resistor being calibrated. Therefore, the detector need not have a very great range, but must have very low noise, high resolution, and high accuracy. Furthermore, since the detector is only measuring small deviations from zero, the linearity of the detector over large ranges is not crucial. In the measurement system described in this paper, the DVM is used to measure voltages that differ widely from zero, and that are both positive and negative. This requires the DVM to have a very high degree of linearity.

Offsets and nonlinear responses of the DVM can be determined by calibrating the DVM against a Josephson array. The Josephson array produces a time-invariant voltage that is related to fundamental constants, and, by international agreement, provides a practical metrological representation of the volt. If the Josephson array produces a defined voltage V, the voltage indicated by the DVM will be:

$$V_{\mathrm{DVM}}=A+gV+N(V),$$ (17)

where *A* is the offset, *g* is the gain of the DVM, and *N*( V) is a nonlinear correction. For most modern high quality meters,

g=1+S, (18)

where *S* is a small number. The values of *A*, *g*, and *N* should be determined by measuring *V*_DVM_ with applied array voltages in the neighborhood of me values expected to be encountered in the resistance measurements. A least-squares fitting procedure should be used to determine the gain, offset, and nonlinear corrections for *both* positive and negative voltages.

If the offset voltage *A* is the same for both positive and negative voltages, then the same averaging procedure that eliminates the thermal voltages will eliminate the offset voltage: the offset will cancel when *V*(*+I*) and *V*(−*I*) are subtracted, as in Eq. (8). In practice, however, neither *A* nor *g* need be the same for positive and negative voltages.

As described in Sec. 2, the ratio of the resistor values is determined from the arithmetic mean of the ratios of the averaged voltages across each resistor in the standard and interchanged position, as in Eq. (14). Each voltage in Eq. (14) must be corrected for the offset, nonunity gain, and nonlinearities in the DVM before the ratios are taken. A factor taking all of these corrections into account can be derived for Eq. (14) as follows: the voltage indicated by the DVM when it is connected to the Hall resistor in the bottom position in the circuit of Fig. 1, with positive current, is given by (neglecting thermal voltages)

$$V_{H}^{\mathrm{BOT}}(+I)=A^{+}+g^{+}\;V_{H}^{\mathrm{BOT}}+N\left(V_{H}^{\mathrm{BOT}}\right),$$ (19)

where $V_{H}^{\mathrm{BOT}}$ is the “true” voltage across the Hall resistor in the bottom position in the circuit of Fig. 1. Likewise, with the current reversed,

$$V_{H}^{\mathrm{BOT}}(-I)=A^{-}-g^{-}V_{H}^{\mathrm{BOT}}+N\left(-\;V_{H}^{\mathrm{BOT}}\right),$$ (20)

Averaging as in Eq. (8) gives:

$$\langle V_{H}^{\mathrm{BOT}}\rangle =\alpha +\gamma V_{H}^{\mathrm{BOT}}+\delta N_{H}^{\mathrm{BOT}},$$ (21a)

where

$$\alpha =\frac{A^{+}-A^{-}}{2},$$ (21b)

$$\gamma =\frac{g^{+}+g^{-}}{2},$$ (21c)

and

$$\delta N_{H}^{\mathrm{BOT}}=\frac{N\left(V_{H}^{\mathrm{BOT}}\right)-N\left(-V_{H}^{\mathrm{BOT}}\right)}{2}.$$ (21d)

If leakage resistances and other systematic effects are ignored, the ratio of the “true” voltages across the resistors is equal to the ratio of the resistors, i.e.,

$$\frac{1}{2}\left(\frac{V_{R}^{\mathrm{TOP}}}{V_{H}^{\mathrm{BOT}}}+\frac{V_{R}^{\mathrm{BOT}}}{V_{H}^{\mathrm{TOP}}}\right)=\frac{G_{H}}{G_{S}}=\frac{R_{S}}{R_{H}}.$$ (22)

Inverting Eq. (21a) above gives

$$V_{H}^{\mathrm{BOT}}=\frac{\langle V_{H}^{\mathrm{BOT}}\rangle -\alpha -\delta N_{H}^{\mathrm{BOT}}}{\gamma }.$$ (23)

Repeating the calculation for each of the voltages and substituting into the left-hand side of Eq. (22) yields

$$\begin{array}{l} \frac{1}{2}[\frac{\left(\frac{\langle V_{R}^{\mathrm{TOP}}\rangle -\alpha -\delta N_{R}^{\mathrm{TOP}}}{\gamma }\right)}{\left(\frac{\langle V_{H}^{\mathrm{BOT}}\rangle -\alpha -\delta N_{H}^{\mathrm{BOT}}}{\gamma }\right)}\\ \frac{\left(\frac{\langle V_{R}^{\mathrm{BOT}}\rangle -\alpha -\delta N_{R}^{\mathrm{BOT}}}{\gamma }\right)}{\left(\frac{\langle V_{H}^{\mathrm{TOP}}\rangle -\alpha -\delta N_{H}^{\mathrm{TOP}}}{\gamma }\right)}]=\frac{R_{S}}{R_{H}}. \end{array}$$ (24)

Generally, the corrections *α* and δ*N* are very much smaller than the values of the voltages 〈*V*〉, so that the ratio of *α+*δ*N* to 〈*V*〉 is of such small magnitude that terms of second order in this quantity can be neglected. Equation (24) can then be simplified to

$$\begin{array}{l} \frac{R_{S}}{R_{H}}=\frac{1}{2}\left(\frac{\langle V_{R}^{\mathrm{TOP}}\rangle }{\langle V_{H}^{\mathrm{BOT}}\rangle }+\frac{\langle V_{R}^{\mathrm{BOT}}\rangle }{\langle V_{H}^{\mathrm{TOP}}\rangle }\right)+\\ \frac{1}{2}[\left(\frac{\langle V_{R}^{\mathrm{TOP}}\rangle }{\langle V_{H}^{\mathrm{BOT}}\rangle }\right)\left(-\frac{\alpha +\delta N_{R}^{\mathrm{TOP}}}{\langle V_{R}^{\mathrm{TOP}}\rangle }+\frac{\alpha +\delta N_{H}^{\mathrm{BOT}}}{\langle V_{H}^{\mathrm{BOT}}\rangle }\right)+\\ \left(\frac{\langle V_{R}^{\mathrm{BOT}}\rangle }{\langle V_{H}^{\mathrm{TOP}}\rangle }\right)\left(-\frac{\alpha +\delta N_{R}^{\mathrm{BOT}}}{\langle V_{R}^{\mathrm{BOT}}\rangle }+\frac{\alpha +\delta N_{H}^{\mathrm{TOP}}}{\langle V_{H}^{\mathrm{TOP}}\rangle }\right)]. \end{array}$$ (25)

The first term on the right side of Eq. (25) is the ratio of the voltages across each resistor [averaged as in Eq. (3)], while the second term on the right side of Eq. (25) is the correction factor that must be applied to correct for nonzero offsets and nonlinearities in the DVM. This expression can be considerably simplified by noting that both *α* and δ*N* are very much smaller than the voltages 〈*V*〉, so the denominators can be replaced by their nominal values. In addition, the δ*N* are the *differences* between the nonlinearity corrections for positive and negative voltages, and since the magnitudes of the voltages across a given resistor are all essentially the same, little error will be incurred by making the approximation that δ*N* is a constant for each resistor. Thus, with the approximations

$$\begin{array}{l} \langle V_{R}^{\mathrm{TOP}}\rangle \approx \langle V_{R}^{\mathrm{BOT}}\rangle =\langle V_{R}\rangle \\ \langle V_{H}^{\mathrm{BOT}}\rangle \approx \langle V_{H}^{\mathrm{TOP}}\rangle =\langle V_{H}\rangle \\ \delta N_{R}^{\mathrm{TOP}}\approx \delta N_{R}^{\mathrm{BOT}}\approx \delta N_{R}\\ \delta N_{H}^{\mathrm{BOT}}\approx \delta N_{H}^{\mathrm{TOP}}\approx \delta N_{H}\\ \frac{\langle V_{R}^{\mathrm{TOP}}\rangle }{\langle V_{H}^{\mathrm{BOT}}\rangle }\approx \frac{R_{S}}{R_{H}}, \end{array}$$ (26)

the correction term in Eq. (25) can be written

$$\frac{1}{\langle V_{H}\rangle }\left(\frac{R_{S}}{R_{H}}(\alpha +\delta N_{H})-(\alpha +\delta N_{R})\right).$$ (27)

It is important to remember that *α* is the *difference* between the offsets for positive and negative voltages, as given by Eq. (21) above. Usually, the offsets are negligibly small and are the same for positive and negative voltages, so that *α* in the above equation can be neglected. In this case, Eq. (27) can be written

$$\frac{1}{\langle V_{H}\rangle }\left(\frac{R_{S}}{R_{H}}\delta N_{H}-\delta N_{R}\right).$$ (28)

The quantity δ*N* in the above equation is the *difference* between the nonlinearity correction for positive voltage and for negative voltage. This need not be zero and must be determined by calibrating the meter against a Josephson array. It should be noted that if both resistors have nominally the same value, then even though δ*N*_R_ and δ*N*_R_, may not be zero, they will have essentially the same value, and the correction term in Eq. (28) will vanish. Corrections for the nonlinearity of the meter therefore need only be made in the case that the resistors being compared have nominally different values. In the case that the ratio of the resistances is 4, this correction term can be quite significant. Using the DVM calibration data in Fig. 2 of Cage et al. [6], and assuming *R*_S_=25 812.807 Ω and *R*_H_=6 453.201 75 Ω, the correction term of Eq. (28) would be 0.40 ppm ± 0.28 ppm. It is important to note that the correction to the value of the resistor *R*_S_, expressed as a relative deviation from its nominal value, $(R_{S}-R_{S}^{\mathrm{nom}})/R_{S}^{\mathrm{nom}}$, due to nonlinearities in the DVM is obtained by dividing Eq. (28) by the ratio $R_{S}^{\mathrm{nom}}/R_{H}$ (sec Sec. 2), which in the case of this example is 4. The correction to the value of $(R_{S}-R_{S}^{\mathrm{nom}})/R_{S}^{\mathrm{nom}}$ due to the DVM nonlinearity is therefore only 0.1 ppm ± 0.07 ppm in this example.

Since the nonlinearity correction term above may change with time, the DVM should be calibrated before each resistance calibration is performed. It should be noted that if either the offsets or the gain vary randomly with time, they will not cancel in the above equations, and will give rise to an uncertainty in the final determination of the value of the standard resistor as discussed in Sec. 4.

#### 3.3.2 Internal Current Source

Modern digital voltmeters inject a small current into the circuit to which their input terminals arc attached. This current is often very small: for a Hewlett Packard 3458A DVM^2^ it was measured to be 10^−14^ A (with no bias applied to the meter input terminals) by connecting a Keithley 602 electrometer directly across the input terminals. If this current does not change sign or magnitude with varying applied bias, it should have no effect on the average voltage measurements, as any offset produced by it would cancel when the voltages measured with positive and negative current are averaged. If it changes sign and magnitude with changing applied bias, but generally retains a magnitude in the range of 10^−14^ A, and if the measurement current is in the range of 10 μA to 50 μA, this small current would result in an error in the ratio of the resistors of only 0.0002 ppm to 0.0010 ppm.

#### 3.3.3 Input Impedance

Whenever a DVM with finite input impedance is connected across one of the resistors, some current will be shunted through the DVM. If the input impedance of the DVM is Z_DVM_=1/*G*_DVM_, and if the admittance of the current source is negligibly small (that is, the current source is assumed to be ideal), then the correction to the resistance ratio with the resistors in the standard position will be:

$$\frac{\langle V_{R}^{\mathrm{TOP}}\rangle }{\langle V_{H}^{\mathrm{BOT}}\rangle }=\frac{G_{H}+G_{\mathrm{DVM}}}{G_{R}+G_{\mathrm{DVM}}}=\frac{G_{H}}{G_{R}}\left(1+\frac{G_{\mathrm{DVM}}}{G_{H}}-\frac{G_{\mathrm{DVM}}}{G_{R}}\right)=\frac{R_{S}}{R_{H}}\left(1+\frac{R_{H}-R_{S}}{Z_{\mathrm{DVM}}}\right).$$ (29)

In the event that the two resistors being compared are of nominally equal value, the correction factor in Eq. (29) will vanish. If however, the resistors have different nominal values, the factor must be evaluated, and can be quite appreciable, particularly if the input impedance of the DVM is less than 10^12^ Ω. In this case, if *R*_S_*−R*_H_(*i*=l)=25 812.807 Ω and *R*_H_(i=4)=6 453.201 75 Ω (a 4-to-l resistance ratio) the correction to the value of *R*_S_ will be as much as 0.019 ppm. In practice, the input impedances of modern DVMs are somewhat higher than 10^12^ Ω. The input impedances of two Hewlett Packard 3458A DVMs were measured using a method described by Cage et al. [6]. The impedances, measured at 22 °C with a relative humidity of 43%, were 5.9×10^12^ Ω for one DVM and 3.6×10^12^ Ω for the other DVM. Such input impedances would lead to only a 0.001 ppm correction to the resistance ratio when calibrating a 10 kΩ resistor against *R*_H_(*i*=4)=6 453.201 75 Ω, and a 0.005 ppm correction to the resistance ratio when calibrating a 10 kΩ resistor against *R*_H_(*i*=1)=25 812.807 Ω.

## 4. Uncertainties Arising from Random Effects

The discussions in the previous sections have concerned only errors and sources of uncertainty in the determination of the value of the standard resistor due to systematic effects. As can be seen from the summary in Table 2, the errors associated with most of these systematic effects can be reduced to the level of 0.01 ppm by appropriate design of the measurement system. Random variations in the various parts of the measurement system, such as nonlinear drifts in the current, thermal voltages, contact resistances, and even the offset and gain of the DVM can also contribute to the uncertainty in the determination of the value of the standard resistor. Many of these have been discussed in Sec. 3 and their effects are summarized in Table 3. Appropriate design and construction of the measurement system, as noted in Sec. 3, generally results in contributions to the combined standard uncertainty from these effects of a few parts in 10^8^. External noise picked up by the cables in the measurement system, and circulating currents arising from multiple ground connections in the circuit (commonly referred to as “ground loops”) can also contribute to the combined standard uncertainty. Fortunately, the influence of these effects on the measurement can be greatly reduced by adequately shielding the measurement cables and equipment, preferably with two layers of shielding, and checking the circuit to ensure that there is only a single connection to ground (sec Ref. [5]). Often this latter task is complicated considerably by the fact that some instruments have various internal connections to ground that are not obvious, so that while it may appear that the meter is isolated from ground, it in fact is cither connected internally to ground through its power cable, or through the chassis, which may be screwed to a rack, which itself may be grounded.

Even if the effects of external noise, random drifts in the system, etc., can be eliminated, the fundamental factor limiting the accuracy of this technique is the internal noise and resolution of the DVM. Modern commercial 8 1/2 digit multimeters are available that have resolutions as fine as 10 nV out of 1 V, as mentioned in Sec. 3.3, so the DVM resolution does not really limit the accuracy of this technique. The internal noise in the DVM, however, can have rms values as high as 0.04 ppm to 0.1 ppm. This, coupled with random, long-term drifts in the gain, offset, and nonlinearity, are primarily responsible for limiting the accuracy of this technique.

In order to minimize the effect of such noise on the measurement, we must first examine the nature of the noise and how it affects the measurement. What we measure are the voltages across the resistors. These may be regarded as fixed voltages, upon which are super-imposed noise voltages, which can have different frequencies and amplitudes. Very high frequency noise, with a mean period less than the time required for a single measurement, will not greatly affect the measurement, for it will average to zero during the measurement time. Thus, the uncertainties in the voltage measurements in Eq. (3) can be greatly reduced by averaging each voltage measurement for a long time. This can be done by making repeated measurements of each voltage over an extended period of time and then averaging these measurements to produce a mean voltage. The uncertainty in this mean will be given by [see Ref. 14]

$$s=\sqrt{\frac{\sum_{i=1}^{N}{\left(V_{i}-\langle V\rangle \right)}^{2}}{N(N-1)}}$$ (30)

It would appear from Eq. (30) that the more measurements of each voltage are made, the smaller the uncertainty in the mean. This, however, is not the case, for in addition to the obvious high-frequency noise that gives rise to scatter in the individual measurements (each of which are assumed to be made over a short time interval of a second or less), there are long term drifts in the current, thermal voltages, and components of the measurement system. These drifts can be considered to be “noise” with very low frequency. In addition, there may be long-term, predictable drifts in the system: an example of this would be the slow, linear decrease in current produced by a mercury battery-powered current source as the batteries get depleted. Averaging a voltage measurement for longer periods of time will decrease the effects of higher frequency noise with periods less than the measurement time, but if these long-term linear drifts in current (for example) are present, there is some measurement time beyond which the effects of short term noise become negligible, and the primary contribution to the uncertainty given by Eq. (30) will be the linear drift in measurement current. For a drift of about 0.2 ppm in 10 min. this time can be as short as 10 h or as long as several days, depending on the magnitude of the noise voltage.

For optimum results, the individual voltage measurements indicated in Eq, (3) should be averaged for short enough times that long term drifts do not contribute to the uncertainty of the measurement at all. Then, as was shown in Sec. 3.2, appropriate averaging of sets of such measurements causes the effect of the long-term drifts to vanish. With modern 8 1/2 digit DVMs, averaging each of the voltage measurements in Eq. (3) for a period of about a minute results in uncertainties in the determination of the mean value of each voltage of between 5 nV and 15 nV, depending on the internal noise of the DVM. This results in a relative standard uncertainty in the determination of the resistance ratio from a single group of measurements of between 0.015 ppm and 0.046 ppm.

It is important to understand that this estimate of the uncertainty in the resistance ratio only considers the contributions from the noise, and does *not* include other random effects, such as irreproducible contact resistances, and various random drifts in the system. Therefore, while the uncertainty due to noise may be quite small, the *actual* value of the ratio may be considerably different from the value determined from a single group of measurements. In order to reduce the combined uncertainty due to all random effects in the determination of the resistance ratio, it is therefore necessary to perform numerous groups of measurements, and then average the resistance ratios r determined from each group to obtain a final average < *r*_i_ >:

$$<r>\;=\frac{1}{N}\sum_{i=1}^{N}r_{i}.$$ (31)

Because long-term drifts and other random effects tend to cause fairly large fluctuations in the values of the *r_i_*, determined from different groups (usually larger than the uncertainty in each *r_i_*, due to noise), the uncertainty in the final resistance ratio determined from Eq. (31) is determined from the set of group means < *r_i_* >

$$s=\sqrt{\frac{1}{N(N-I)}\sum_{i=1}^{N}{\left(r_{i}-<r>\right)}^{2}}.$$ (32)

The uncertainty calculated from Eq. (32) includes the effects of noise, and of random variations in the system, so there is no need to include these effects explicitly. The magnitude of the uncertainty will depend on the magnitudes of these various random effects. When the internal noise in the DVM is sufficiently low that each voltage measurement has an uncertainty on the order of 5 nV to 7 nV, noise in the DVM will actually be only a minor factor contributing to the final uncertainty; more significant contributions will come from the irreproducibility of the contact resistances and other factors shown in Table 3. If these random effects are kept within the limits shown in Table 3, however, it is usually possible to obtain relative uncertainties (due to random effects) in the determination of the mean resistance ratio [Eq. (31)] of less man 0.01 ppm after 20 groups of measurements.

## 5. Conclusion

Standards laboratories requiring resistance calibrations with relative combined standard uncertainties of less than 0.1 ppm could benefit from a simple, inexpensive, intrinsic resistance standard. While the quantum Hall effect provides such a standard of resistance, the measurement systems used at most national standards laboratories are far too expensive, complex, and time consuming to construct and use in government and industrial standards laboratories. This paper has analyzed the sources of uncertainty arising from both systematic and random effects in a simple quantized Hall resistance measurement system that uses a modern commercially available 8 1/2 digit digital voltmeter to compare the voltages developed across a standard resistor placed in series with a quantized Hall resistor. A measurement sequence has been presented which minimizes the effects of thermal voltages and linear drifts in the current on the final determination of the unknown resistance. Criteria have also been presented for minimizing the effects of the contact resistances and leakage resistances.

Table 2 summarizes the systematic effects that cause errors in the determination of the resistance using this technique. Most of these errors are so small as to be negligible in comparison with the uncertainty in the Final resistance ratio due to random effects, and these corrections arc therefore neglected and for simplicity are included in the uncertainty of the final result. In the case of the nonlinearity of the DVM, however, the error can be fairly large, and a correction factor [derived in Eq. (25)] must be applied to the final result Table 3 summarizes the permissible magnitudes of random variations and drifts in the various components of the measurement system.

The combined standard uncertainty in the value of the standard resistor is the square root of the sum of the squares of the standard uncertainties arising from both random and systematic effects in the voltage measurements. If the uncertainty from random effects is less than 0.06 ppm (easily achievable with just a single group of measurements), the relative combined standard uncertainty in the determination of the resistance can be as low as 0.06 ppm if the DVM nonlinearity correction in Table 2 is negligible (including for simplicity the correction factors in Table 2 as sources of uncertainty). If the DVM nonlinearity correction is as high as that shown in Table 2, the combined uncertainty will be as high as 0.09 ppm.

If the uncertainty due to random effects is less than 0.01 ppm, which can be achieved by averaging up to 20 groups of data as described in Sec. 4, the relative combined standard uncertainty in the value of the standard resistor can be less than 0.03 ppm (again assuming the DVM nonlinearity correction to be negligible, as would be the case if the resistors being compared had the same nominal value). This DVM-based measurement system can, therefore, be used to compare wire-wound standard reference resistors with quantum Hall resistors with a relative combined uncertainty of less than 0.1 ppm, and in the most favorable cases, with uncertainties less than 0.06 ppm. Since the quantized Hall resistance does not drift with time, this measurement system can be used to calibrate wire-wound resistors, the values of which tend to drift with time.

## Figures and Tables

**Table 1:** Assuming a constant rate of change in the current produced by the current source and that each voltage measurement takes a time Δ*t*, the current is calculated at the beginning of each voltage measurement on the standard resistor (*R*_S_) and and the Hall resistor (*R*_H_). Each set of four measurements on a resistor is averaged 10 eliminate thermal voltages and other constant offsets, resulting in the average voltages shown in column 3.

Resistor	*I*	Mean voltage
*R*_s_(+) *R*_s_(−) *R*_s_(−) *R*_s_(+)	$\begin{array}{c} +I_{0}\\ -(I_{0}-B\Delta t)\\ -(I_{0}-2B\Delta t)\\ +(I_{0}-3B\Delta t) \end{array}\}$	$R_{S}\left[I_{0}-\left(\frac{3}{2}\right)B\Delta t\right]$
*R*_H_(+) *R*_H_(−) *R*_H_(−) *R*_H_(+)	$\begin{array}{c} +(I_{0}-4B\Delta t)\\ -(I_{0}-5B\Delta t)\\ -(I_{0}-6B\Delta t)\\ +(I_{0}-7B\Delta t) \end{array}\}$	$R_{H}\left[I_{0}-\left(\frac{11}{2}\right)B\Delta t\right]$
*R*_H_(+) *R*_H_(−) *R*_H_(−) *R*_H_(+)	$\begin{array}{c} +(I_{0}-8B\Delta t)\\ -(I_{0}-9B\Delta t)\\ -(I_{0}-10B\Delta t)\\ +(I_{0}-11B\Delta t) \end{array}\}$	$R_{H}\left[I_{0}-\left(\frac{19}{2}\right)B\Delta t\right]$
*R*_s_(+) *R*_s_(−) *R*_s_(−) *R*_s_(+)	$\begin{array}{c} +(I_{0}-12B\Delta t)\\ -(I_{0}-13B\Delta t)\\ -(I_{0}-14B\Delta t)\\ +(I_{0}-15B\Delta t) \end{array}\}$	$R_{S}\left[I_{0}-\left(\frac{27}{2}\right)B\Delta t\right]$

**Table 2:** Systematic effects that give rise to corrections to the value of the standard resistor determined using this method. If the value of each effect is kept within the limits shown in the second column, the value of the correction to the deviation of the value of *R*_S_ from Us nominal value, given by the quantity $(R_{S}-R_{S}^{\mathrm{nom}})/R_{S}^{\mathrm{nom}}$, will be shown in the third column. The first value was calculated assuming that *R*_S_=10 kΩ and *R*_H_(*i*=4)=6 453.201 75 Ω; the second value was calculated assuming *R*_S_=10 kΩ and *R*_H_=25 812.807 Ω. Since the values of these corrections are generally very small in comparison with the uncertainty due to random effects, these correction factors are not applied, but for simplicity arc included in the uncertainty of the final result.

Effect	Value	Absolute value of correction to result (in ppm)	Section in which calculated:
Leakage resistance	>10^12^ Ω	0.004 to 0.016	3.1.3
DVM input impedance	>10^12^ Ω	<0.003 to 0.015	3.3.3
DVM nonlinearity	<0.05 μV ±0.01 μV	0 to <0.10±0.07	3.3.1

**Table 3:** Randomly varying effects that contribute to the uncertainty in the determination of the value of the standard resistor In the first row, the first number in the third column was calculated assuming *R*_S_=10 Ω and *R*_H_=6 453.201 75 Ω; the second number was calculated assuming *R*_H_=25 812.807 Ω.

Effect	Value	Standard uncertainty (in ppm)	Section in which calculated
Contact resistance reproducibility	<0.001 Ω	0.017 to 0.046	3.1.2
Nonlinear drifts in current source	<10^13^ A	<0.005	3.2
DVM current source	<10^−13^ A	0.000 to 0.001	3.3.2
Noise in DVM	5nV to 15 nV for 30 s measurements	0.015 to 0.046 for single group of measurements	4

## Footnotes

1 The current source is represented by its Norton equivalent, i.e.. an ideal current source *I*_o_ in parallel with an equivalent internal admittance *Y*_c_. See Ref. [10] for more information.

2 Certain commercial equipment, instruments, or materials are identified in this paper in order to adequately specify the experimental procedure. Such identification does not imply recommendation or endorsement by the National Institute of Standards and Technology, nor does it imply that the materials or equipment identified are necessarily the best available for the purpose.
